# Equine tendonitis therapy using mesenchymal stem cells and platelet concentrates: a randomized controlled trial

**DOI:** 10.1186/scrt236

**Published:** 2013-07-22

**Authors:** Armando de Mattos Carvalho, Peres Ramos Badial, Luis Emiliano Cisneros Álvarez, Ana Lucia Miluzzi Yamada, Alexandre Secorun Borges, Elenice Deffune, Carlos Alberto Hussni, Ana Liz Garcia Alves

**Affiliations:** 1 Department of Veterinary Surgery and Anesthesiology, School of Veterinary Medicine and Animal Science, São Paulo State University, Rubião Junior, 18618-970 Botucatu, São Paulo,Brazil; 2 Department of Veterinary Clinic, School of Veterinary Medicine and Animal Science, São Paulo State University, Rubião Junior, 18618-970 Botucatu, São Paulo, Brazil; 3 Department of Animal Reproduction and Veterinary Radiology, School of Veterinary Medicine and Animal Science, São Paulo State University, Rubião Junior, 18618-970 Botucatu, São Paulo, Brazil; 4 Blood Center, Botucatu Medical School, São Paulo State University, Rubião Junior, 18618-970 Botucatu, São Paulo, Brazil

**Keywords:** Tendon lesion, Treatment, Stem cell, Horse

## Abstract

**Introduction:**

Tendon injury is a major cause of lameness and decreased performance in athletic equines. Various therapies for tendonitis have been described; however, none of these therapies results in complete tissue regeneration, and the injury recurrence rate is high even after long recovery periods involving rest and physiotherapy.

**Methods:**

A lesion was induced with collagenase gel in the superficial digital flexor tendon in the center portion of the metacarpal region of eight equines of mixed breed. After two weeks, the lesions of the animals in the treated and control groups were treated through the intralesional administration of mesenchymal stem cells derived from adipose tissue (adMSCs) suspended in platelet concentrate (PC) and with phosphate buffered saline (PBS), respectively. Serial ultrasound analyses were performed every two weeks. After 16 weeks of therapy, a biopsy was performed for histopathological, immunohistochemical and gene expression (type I collagen (*COL1A1*), type III collagen (*COL3A1*), tenascin-C (*TNC*), tenomodulin (*TNMD*), and scleraxis (*SCX*)) analyses.

**Results:**

Differences in the ultrasound and histopathological analyses were observed between the groups. Improved results were reported in the group treated with adMSCs suspended in PC. There was no difference in the gene expression levels observed after the different treatments. The main results observed from the histopathological evaluation of the treated group were as follows: a prevention of the progression of the lesion, a greater organization of collagen fibers, and a decreased inflammatory infiltrate. A lack of progression of the lesion area and its percentage was observed in the ultrasound image, and increased blood flow was measured by Power Doppler.

**Conclusions:**

The use of adMSCs combined with PC for the therapy of experimentally induced tendonitis prevented the progression of the tendon lesion, as observed in the ultrasound examination, and resulted in a greater organization and decreased inflammation, as observed in the histopathological evaluation. These data demonstrate the therapeutic potential of this therapy for the treatment of equine tendonitis.

## Introduction

Tendon lesions are a major cause of lameness and reduced performance in athletic horses and may result in the early termination of the animals’ careers [1]. Tendinous structures are poorly vascularized, are relatively acellular, and have limited potential for regeneration because the tendon repair is prolonged and results in the formation of scar tissue, which is biomechanically inferior and prone to lesion recurrence [2].

A tendon injury usually requires a considerable rest period (from six months to over a year) to allow adequate tissue repair. Conventional treatments for tendonitis are basically clinical and surgical approaches. However, the recovered animals are rarely sufficiently healed to allow their return to competitions with a performance similar to that observed when they were healthy [3].

Regenerative medicine has significantly evolved. In addition, the use of mesenchymal stem cells (MSCs) and the implantation of platelet concentrate (PC) in the therapy of equine tendonitis have been previously described [4-14]. Previous studies have shown a significant decrease in the recurrence rate of tendonitis for a period of at least two years in animals treated with cell therapy (27%) compared with those that were treated with conventional therapies (56%) [15].

There are several sources of MSCs available, including the bone marrow, the adipose tissue, the peripheral blood, the blood and matrix from the umbilical cord, and the tendons [16]. Of these sources, the bone marrow has been considered the most important source of MSCs. However, other sources, such as the adipose tissue, exhibit interesting characteristics, such as a greater number of viable MSCs with a high rate of migration and cell differentiation; thus, these characteristics make this source a convenient source of progenitor cells [17].

PC is an autologous biological product that is used in equine sports medicine with evident clinical efficacy. The benefits of this therapy include minimally invasive collection and fast, easy, and low-cost processing. The therapeutic efficacy of PC is due to the release of several growth factors that are injected directly into the injured area [18]. The studies that demonstrate the potential of PC in the therapy of equine tendonitis, such as those that analyzed the improvement of the score obtained through the ultrasound evaluation of clinical cases [14], showed increased local vascularization [19] and metabolic activity, which resulted in the maturation of tissue repair [12].

The suspension of MSCs in PC has been used with promising results in bone repair [20-22]. Only one clinical study in equines investigated equine tendonitis therapy using mesenchymal stem cells derived from adipose tissue (adMSCs) suspended in PC [11], and 14 out of the 16 animals that underwent superficial digital flexor tendon (SDFT) therapy returned to athletic activity.

The present study aimed to evaluate the effects of autologous adMSCs suspended in autologous PC in the therapy of animals with experimentally induced tendonitis through ultrasound, histopathological, immunohistochemical and gene expression analyses.

## Methods

### Animals

Eight young horses (seven females and one male) of mixed breed and aged between three and four and a half years were used. The animals were clinically and ultrasonographically examined before the start of the experiment to ensure that there was no tendon injury. A complete blood count was also performed to confirm the health status of the animals. All of the animals were placed in individual stalls and adapted to daily management for 21 days before the start of the experiment. The protocol was approved by the ethics committee for animal experimentation of the university (number: 213/2008). In addition to the eight tendons of the horses used in the experiment, four tendons were used that were obtained from equines (two males and two females, mixed breed, aged between three and eight years) that were euthanized due to complications during surgical procedures that did not compromise the SDFT. The samples from euthanized horses were used to standardize the quantitative reverse transcription polymerase chain reaction (qRT-PCR) analysis. The animals’ previous histories of tendonitis were also researched to ensure that the collected fragments were obtained from healthy tendons.

### Study design

**Figure 1:**
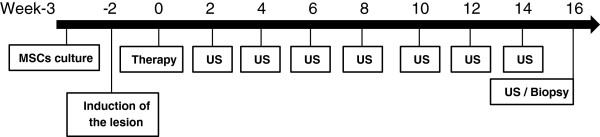
**Timeline.** US, ultrasound.

The study consisted of two randomly chosen groups: the treated group (TG; tendon treated with adMSCs, n = 4) and the control group (CG; tendon treated with PBS, n = 4). The collection of adipose tissue was performed three weeks before the administration of the therapy in order to obtain an adequate number of MSCs in culture (Week −3). The tendon lesion was then induced two weeks before the beginning of the therapy. This time is enough to consider a consistent lesion at an early stage (Week −2). Ultrasound examinations were performed every two weeks until the end of the experiment. After 16 weeks of therapy, a biopsy of the SDFT was performed for histopathological, immunohistochemical and gene expression evaluations (Week 16). All of the exams were performed by experimenters blinded to the study conditions (Figure 1).

Samples from the SDFT of four euthanized horses with no history of tendonitis (HG, healthy group, n = 4) were used to assess the gene expression differences between the TG and the CG.

### Isolation and cultivation of stem cells

For the collection of adipose tissue, the animals were sedated with xylazine (0.7 mg/kg, i.v.). This treatment was followed by trichotomy, antisepsis and the subcutaneous injection of 20 mL of 2% lidocaine hydrochloride using an inverted-L anesthetic block. An approximately 8 cm long incision was made. This incision was approximately 15 cm abaxial to the spine to allow the visualization of a layer of fat between the skin and the musculature. Approximately 2 g of fat was collected per animal. The fat was immediately packed in a tube containing 50 mL of Knockout DMEM (Dulbecco’s modified Eagle’s medium).

The adipose tissue was rinsed with PBS to remove any contamination with whole blood. This step was followed by a gentle mechanical separation using a scalpel and anatomical forceps. Then, 3 mL of type I collagenase diluted in 2 mg/mL Knockout DMEM was added to the mixture. The mixture was then incubated for 12 hours in a humidified incubator at 37°C with 5% CO_2_ for the enzymatic digestion of the adipose tissue. An equal volume of Knockout DMEM containing 10% fetal bovine serum (FBS) was then added to the mixture to neutralize the enzyme action; the mixture was then rinsed with PBS. The cell pellet obtained from each animal was diluted in Knockout DMEM containing 10% FBS and transferred to 75-cm^2^ culture plates, which were placed in an incubator. The culture medium was changed every 48 hours until the culture plate reached 70% confluence. The trypsinization of the cells was then performed, and the cells were subjected to the Trypan blue exclusion test of cell viability. The culture was maintained until the administration of the cell therapy. Prior to the cellular implantation, an aliquot of cells (P1) was collected to perform the immunophenotypic characterization of the cultured cells. As in a previous study, the following markers were used in this characterization: CD44, CD90, CD105 and MHC Class II [23]. Adipogenic, chondrogenic and osteogenic differentiations were also performed to demonstrate the multipotent potential of the cells used (data not shown).

### Induction of the tendon lesion

**Figure 2:**
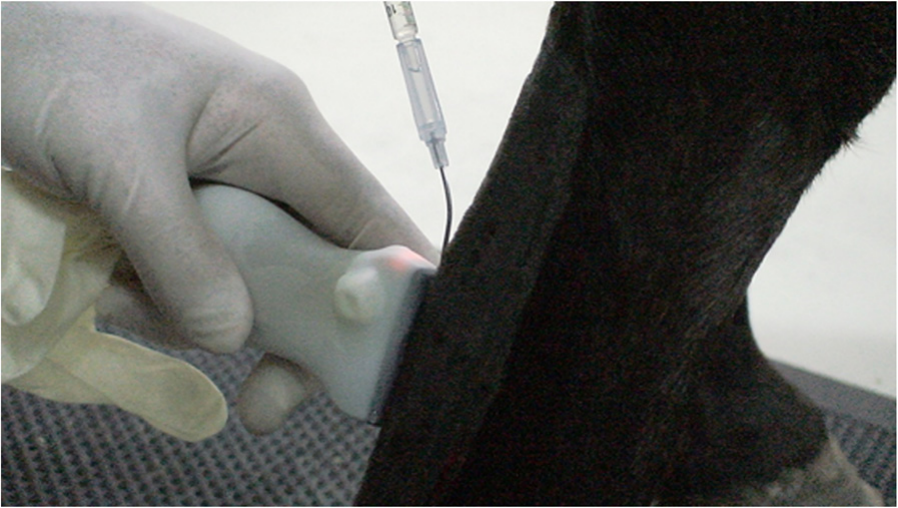
**Lesion induction image.** Lesion induction was guided by an ultrasound device.

The tendon lesion was induced in the middle region of the metacarpus (in the central portion of the SDFT) in one of the forelimbs of the equine, which was randomly selected, using sterile-filtered collagenase type I (Sigma, St. Louis, MO, USA). Then, 1,000 U of gel collagenase was injected into an approximately two-inch-long column defect in the center of the stress region of the SDFT, 10 to 12 cm distal to the accessory carpal bone (ACB). For the correct induction of the lesion, the implantation was guided by an ultrasound device (My Vet Lab 70, Esaote, Genova, Italy) using a sterile 16-gauge (G) catheter needle that was manually curved to a 45° angle (Figure 2) [24]. After the induction of the lesion, the bandage was tied down to the selected member and maintained for one week.

### Cell therapy

Two weeks after the induction of the lesion (T = week 0), the tendon lesions were treated according to the treatment protocol of the respective groups using intralesional injections that were guided by ultrasound. The treated group received 10 × 10^6^ adMSCs, which were suspended in 1 mL of autologous PC. The collection and processing of the whole blood were performed minutes before the cell implantation using a previously established protocol (the platelet activation with calcium hydrochloride was not performed) [25]. The numbers of platelets in the whole blood and PC were counted. Aliquots containing 1 mL of PBS for implantation in the control group were also prepared.

For the administration of the therapy, a local trichotomy was required. This step was followed by anesthetic block with 2 mL of 2% lidocaine hydrochloride at the point of the needle insertion and antisepsis. The animals were also sedated with detomidine (20 μg/kg, i.v.) and maintained in the catch pen throughout the procedure. A 21-G needle was used for the administration of the therapy. The needle was inserted into the center of the SDFT lesion with the aid of ultrasound. The animals remained individually confined throughout the experiment, and the selected members remained bandaged for one week after the therapy.

### Clinical evaluation and physical activity

The vital signs of all of the animals were measured for the evaluation of specific clinical signs, such as local sensitivity, increased temperature and circumference of the central region of the metacarpus. The animals were also examined daily for lameness throughout the experiment, as previously described [26].

•Week 0 to 2: Rest in a stall.

•Week 3 to 5: Walk for 10 minutes, once a day.

•Week 6 to 8: Walk for 10 minutes, twice a day.

•Week 9 to 11: Walk for 20 minutes, once a day.

•Week 12 to 14: Walk for 20 minutes, twice a day.

•Week 13 to 16: Walk for 30 minutes, once a day.

### Ultrasonographic evaluation

Ultrasonographic evaluations were performed prior to the induction of the lesion, before and after the administration of the therapy (Week 0), and every 14 days after the administration of the therapy through the end of the experiment (Weeks 2, 4, 6, 8, 10, 12, 14 and 16). The real-time B-mode examination was performed using ultrasound (My Lab 70, Esaote, Genoa, Italy) equipped with a 3- to-11 MHz multi-frequency linear transducer. The lesion regions that received therapy were also evaluated using Power Doppler to evaluate the micro-vascularization of the tendon with Doppler frequency of 6.7 MHz, pulse repetition frequency (PRF) of 370 Hz, and wall filter of 3.

The tendon region to be treated for each animal was determined immediately before the administration of the therapy, that is, two weeks after the induction of the lesion. The larger cross-sectional lesion area over the approximately 2 cm lesion was used as the criterion.

The B-mode ultrasound was used to collect longitudinal images to evaluate the parallelism of the fibers and to collect cross-sectional images to measure both the cross-sectional tendon area and the cross-sectional lesion area. The percentage of the cross-sectional lesion area (lesion area/tendon area × 100) was also calculated.

The score used to evaluate the bone vascularity was adapted for Power Doppler evaluation (Table 1) [27]. This score classification was used to score the three images obtained in the Power Doppler evaluation videos to evidence the greatest possible blood flow. The mean score obtained for each animal at various time points was calculated, and these data were compared between the different groups.

**Table 1:** Three analyzed categories and their respective scores in the evaluation of the Power Doppler signals

**Category/Score**	**0**	**1**	**2**	**3**
Signal strength	No signal	Red	Orange	Yellow
		Dark Blue	Blue	Peacock Blue
Vessel area (mm^2^)	No signal	1	2 to 3	>3
Number of vessels	No signal	1	2 to 3	>3

The Power Doppler signal strength was observed in different colors due to differences in the blood flow direction.

### Tendon biopsy

The biopsy was performed exactly in the center of the lesion 16 weeks after the start of the therapy. The animals were subjected to inhalational anesthesia and received 10% xylazine (1.1 mg/kg, i.v.) as premedication. Diazepam (0.15 mg/kg, i.v.) and ketamine (2.2 mg/kg, i.v.; Dopalen, Vetbrands, Paulínia, São Paulo, Brazil) were used for the induction, and isoflurane (Cristália Chemicals and Pharmaceuticals, Ltd., Itapira, São Paulo, Brazil) vaporized in 100% oxygen was used for anesthesia maintenance.

The surgical procedures were performed with the animals in right lateral decubitus. An approximately 2 cm long incision was made at the exact point of injury (11 to 12 cm ACB) in the skin and subcutaneous tissue until the SDFT injury was identified. The biopsy was performed in the central portion of the SDFT; the biopsy covered the total area of the tendon lesion. After the correct identification of the injured area, an approximately 0.5-cm^3^ sample of the SDFT was collected and properly stored for subsequent histopathological evaluation, immunohistochemical analysis (in 10% buffered formalin solution), and RT-PCR (frozen in liquid nitrogen) (see Additional file 1: Figure S1). A simple continuous suture of the paratendon was performed using Vicryl 2–0 (Ethicon Inc., Johnson & Johnson Medical Limited, Kirkton Campus, Livingston, West Lothian, UK), followed by skin suture with simple points separated with nylon. After the surgical procedure, a compressive bandage was applied to the metacarpal region. The bandage was changed every 24 hours to dress the wound and was maintained until the removal of the stitches two weeks after the surgery. In the immediate postoperative period, all of the animals were treated with phenylbutazone (4.4 mg/kg, i.v.), which is a non-steroidal anti-inflammatory drug, on the first day and for the next four days (2.2 mg/kg, PO q24 h).

### Histopathological evaluation

Histological sections were prepared in a longitudinal paraffin block that was processed using the conventional paraffinization method. The histological sections were stained with hematoxylin and eosin (H&E) and Masson’s trichrome and examined under a light microscope. The assessor was blinded to the experimental group. Two histological sections were used in each analysis. The histopathological analysis was based on scores. All of the tendon parameters were scored between 1 (normal) and 4 (severe changes): shape of tenocytes, density of tenocytes, hemorrhage, neovascularization, inflammatory cell infiltrate, linearity of collagen fibers, uniformity of collagen fibers and thickness of the epitendon. These assessments were based on those used in previous studies on tendon repair [4,26].

### Immunohistochemical evaluation

The 5-μm-thick cuts obtained from the paraffin blocks were extended on immunoslides (EasyPath, São Paulo, SP, Brazil). Prior to incubation with the primary antibodies for collagen type III and factor VIII, the material was deparaffinized with successive xylene baths and rinsed three times in absolute alcohol followed by washes in solutions with decreasing alcohol concentration (95% and 85%). The antigen retrieval was performed through enzymatic digestion with 2% pepsin (pH 1.8) for 10 minutes in a 60°C incubator and then for 50 minutes in a 37°C incubator. The blocking of the endogenous peroxidase was performed using hydrogen peroxide (3%) in methanol for 20 minutes, followed by 10 washes with distilled water. The peroxidase blocking was followed by the blocking of nonspecific binding with 3% milk powder for 1 h in a 27°C incubator. The slides were subsequently rinsed with TRIS solution. The primary antibodies were incubated for 18 h (overnight) at 6°C using polyclonal anti-bovine antibodies for collagen type III (Novotec, Saint Martin la Garenne, France) and sheep anti-factor VIII (AbD Serotec, Kidlington, Oxford, UK). The slides were incubated with the secondary antibody (EnVision; Dako, Carpinteria, CA, USA) at room temperature for 1 h. The material was then incubated with DAB (Dako) as chromogen at a dilution of one drop per mL of solution. The counter-staining was performed with hematoxylin.

To determine the expression of the markers used, five fields of each fragment, which were given a graduation score ranging from 1 to 4 according to the intensity and abundance of the marking, were assessed (Table 2).

**Table 2:** Histological and immunohistochemical scoring system used to grade tendon repair in equines

**Variable**	**Score and criterion**
Shape of tendon cells	1 = Linear (normal)
	2 = Slightly oval
	3 = Moderately round
	4 = Predominantly round
Density of tendon cells	1 = Sparse (normal)
	2 = Slightly increased
	3 = Moderately increased
	4 = Cell layers
Hemorrhage	1 = Absent (normal)
	2 = Sparse or uneven
	3 = Multiple areas
	4 = Predominantly hemorrhagic
Number of vessels	1 = Normal
	2 = Slightly increased
	3 = Moderately increased
	4 = Severely increased
Inflammatory cell infiltrate	1 = Normal
	2 = Slightly increased
	3 = Moderately increased
	4 = Severely increased
Linearity of collagen fibers	1 = Linear
	2 = >50% linear
	3 = 20% to 50% linear
	4 = Absence of linear areas
Uniformity of collagen fibers	1 = Uniformity of the diameter of all fibers
	2 = > 50% of the fibers are uniform
	3 = 20% to 50% of the fibers are linear
	4 = Complete disorder of the fibers
Collagen type III*	1 = < 10% type III
	2 = 10% to 50% type III
	3 = > 50% a 90% type III
	4 = > 90% type III
Factor VIII*	1 = Average labeling of cells, no recognition of the vascular structure
	2 = Labeling of a few small vessels
	3 = Labeling of several small vessels or greater vessels
	4 = Labeling of several great vessels

*Determined from the immunohistochemical analysis.

### RNA purification and qRT-PCR

The total cellular RNA was purified from tendon tissue samples using QIA shredder columns (QIAGEN Inc., Valencia, CA, USA) and the RNeasy Fibrous Tissue Mini Kit (QIAGEN Inc.). The purity and relative quality of the RNA were determined by spectrophotometry (NanoDrop® 1000 Spectrophotometer, Thermo Scientific™, Wilmington, DE, USA). All of the RNA samples were treated with RQ1 purified RQ1 RNase Free DNase (Promega, Madison, WI, USA) to remove the genomic DNA from the samples. The first-strand cDNA was synthesized using the SuperScript® VILO™ cDNA Synthesis Kit (Invitrogen™, Carlsbad, CA, USA).

The relative quantification of the gene expression in the TG and CG tendons compared with the HG tendon was performed using the comparative Ct method (2^−ΔΔCT^ method) for the relative processing of real-time PCR data [28], which was obtained with a 7500 Fast Real-Time PCR System (Applied Biosystems™, Foster City, CA, USA) and a TaqMan PCR Universal Master Mix II. The equine primers for type I collagen (*COL1A1*), type III collagen (*COL3A1*), tenascin-C (*TNC*), tenomodulin (*TNMD*), scleraxis (*SCX*), *18S* and *β-actin* were designed as previously described [26,29]. *COL1A1, COL3A1, TNC, TNMD* and *SCX* were used as target genes, whereas *18S* and *β-actin* were used as reference genes.

Each RT-PCR reaction was performed in triplicate and conducted in a total volume of 20 μL, which contained 0.3 μM of each forward and reverse primers, 0.15 μM of each probe, 2 μL of the cDNA template, 10 μL of the master mix, and 6.5 μL of nuclease-free water. Additionally, a “no template” control was included in triplicate on each plate. The following PCR conditions were used: initial denaturation at 95°C for 10 minutes and 40 cycles at 95°C for 15 s and 60°C for 1 minute. The data analysis was performed through the normalization of the amplified value of each target gene with the arithmetic mean of the threshold cycle (CT) of the corresponding endogenous control. A sample of the HG was selected for calibration and used on each plate. The gene expression of the target genes in the TG and CG was calculated relative to that in the HG.

### Statistical analysis

For the ultrasonographic evaluation, the mixed model repeated measures design (PROC MIXED, SAS Institute Inc., Cary, North Carolina, USA) was used to compare the average lesion percentage or the average lesion area between the experimental groups and time points. The initial time (Week 0) was also used as a reference for the comparison of the different time points within a group. An interaction term between the treatment and the time point was included to test the hypothesis that the difference between treatments was time-dependent. Tukey’s method was used to adjust the *P*-values that resulted from the multiple comparisons. An autoregressive covariance structure was used to model the repeated measures in the same animal. The Wilcoxon test (PROC NPAR1WAY, SAS Institute Inc., Cary, North Carolina, USA) was used to compare the median score of echogenicity of each time point between treatments.

In the histopathological evaluation, the Wilcoxon test (PROC NPAR1WAY, SAS Institute Inc., Cary, North Carolina, USA) was used to compare the average sum of the scores between the different groups. In the immunohistochemical evaluation, the Wilcoxon test (PROC NPAR1WAY, SAS Institute Inc., Cary, North Carolina, USA) was used to compare the median score between the different treatments. We also used the Wilcoxon test (PROC NPAR1WAY, SAS Institute Inc., Cary, North Carolina, USA) for the statistical evaluation of the relative gene expression of *COL1A1, COL3A1, TNC, TNMD* and *SCX* between the TG and the CG. The Wilcoxon test was also used to compare the RNA concentration obtained between the different groups.

## Results

### Animals

One of the animals of the control group died during the course of the experiment due to causes unrelated to the study; this death decreased the number of animals in our experiment.

### Isolation and cultivation of adMSCs

Approximately 2 g of adipose tissue was collected from the selected animals. No problems were encountered with the healing of the wound located on the back of the animals. A cell cultivation time of three weeks was sufficient for the expansion of the necessary number of adMSCs (10 × 10^6^) for the cell therapy on the first pass (P1). After five days of culture, the number of viable stem cells ranged from 3 × 10^6^ to 6 × 10^6^ (mean = 4 × 10^6^), with a cell viability greater than 96% in all of the samples. After 21 days of cell culture, the number of viable cells ranged from 14 × 10^6^ to 22 × 10^6^ (mean 16 × 10^6^), with a cell viability greater than 98%.

### Cell therapy and clinical evaluation

The implantation of 10 × 10^6^ equine adMSCs suspended in platelet concentrate and the intralesional injection of PBS was successfully performed in all of the animals. The platelet concentration in the whole-blood samples ranged from 110 × 10^3^ platelets/μL to 171 × 10^3^ platelets/μL (mean = 121 × 10^3^ platelets/μL). The autologous platelet concentrate used for the suspension of the MSCs derived from adipose tissue was successfully obtained in all of the samples; the platelet concentration ranged from 305 × 10^3^ platelets/μL to 377 × 10^3^ platelets/μL (mean = 321 × 10^3^/μL). The leukocyte concentration ranged from 2,050 leukocytes/μL to 2,900 leukocytes/μL (mean = 2,350 leukocytes/μL).

There was no change in any of the clinical parameters of the animals during the experiment, and there was no difference in the sensitivity, the temperature and the circumference of the central metacarpal area, as well as the presence of lameness, between the different experimental groups.

### Ultrasonographic evaluation

**Figure 3:**
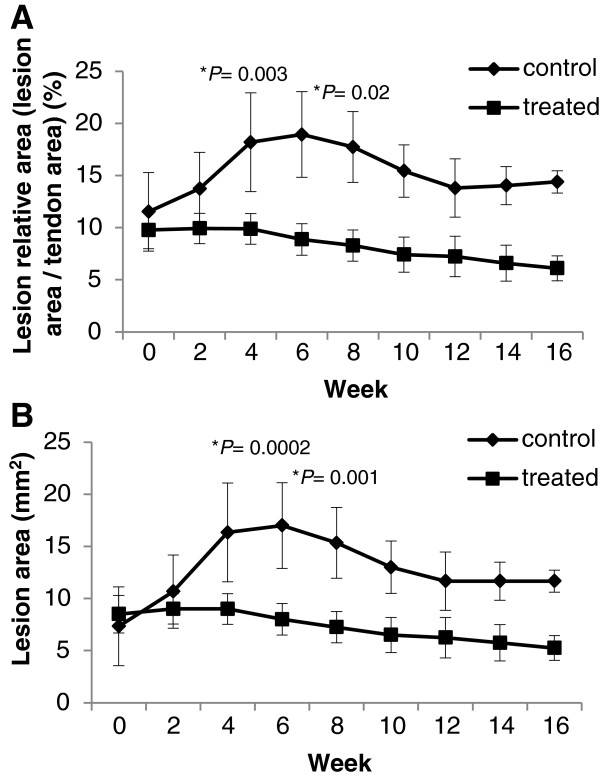
**Ultrasonographic measurements. A)** Relationship between the lesion area in the treated and the control groups at different time points after therapy. **B)** Relationship between the percentage of the cross-sectional lesion area in the different groups at different time points after therapy.

**Figure 4:**
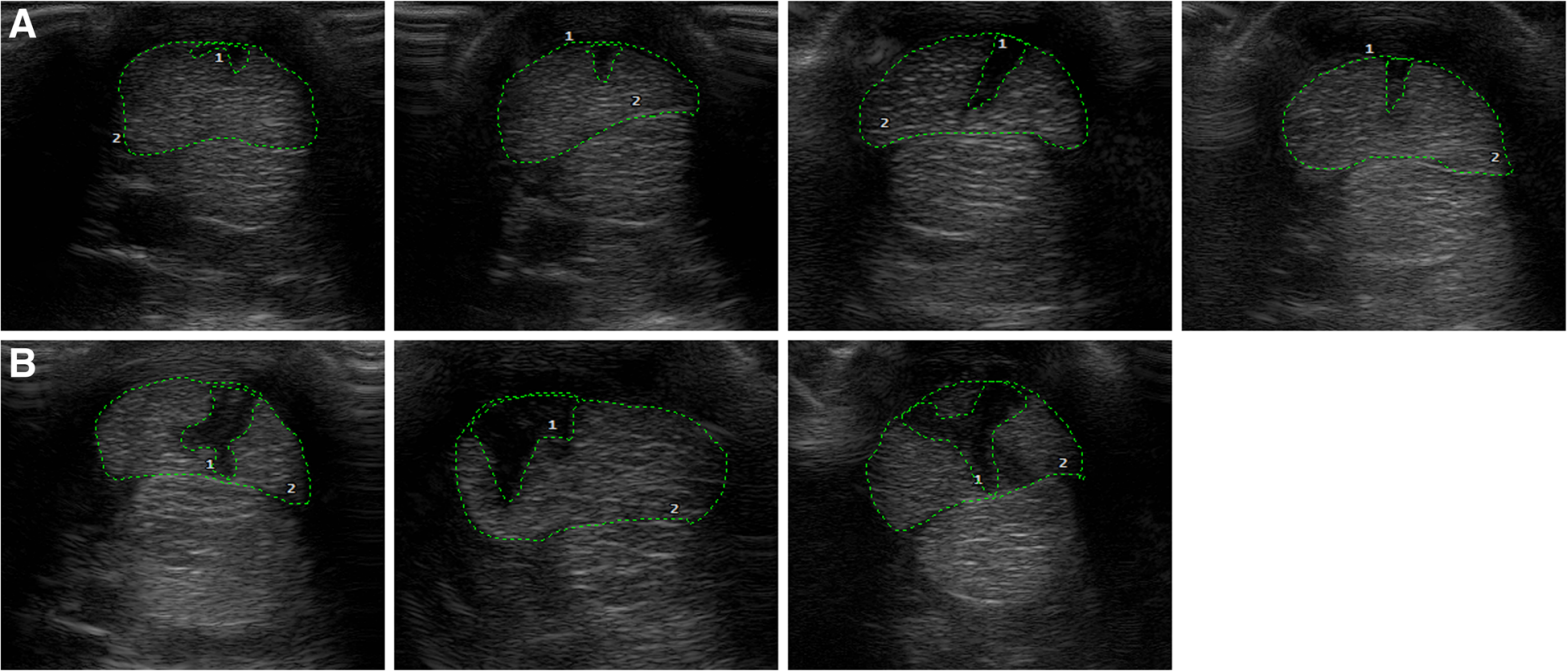
**Transverse ultrasound images.** The images were obtained 12 cm distal to the accessory carpal bone, six weeks after therapy. **A)** Treated group. **B)** Control group.

The results of the statistical modeling suggested that there was a time-dependent difference in the cross-sectional lesion area and its percentage between the treatments (*P* = 0.02 and 0.002 for the interaction between treatments and time points, respectively). However, the comparisons between the treatments made within each time point revealed no significant differences (Figure 3). The figure shows that the cross-sectional lesion area and its percentage in the TG remained constant and then decreased. The figure also shows that there was an increase in the cross-sectional lesion area and its percentage in the CG at Weeks 2, 4 and 6 (Figure 4). This increase was followed by a subsequent decrease. A different data analysis observed the evolution of the cross-sectional lesion area and its percentage in the TG and the CG between the time of treatment administration (Week 0) and the other studied time points. The analysis showed that the CG had a significant difference between Week 0 and Week 4 (cross-sectional lesion area, *P* = 0.0002; percentage of cross-sectional lesion area, P = 0.0003) and between Week 0 and Week 6 (cross-sectional lesion area, *P* = 0.001, percentage of cross-sectional lesion area, *P* = 0.022), whereas the TG did not show any differences (Figure 3).

**Figure 5:**
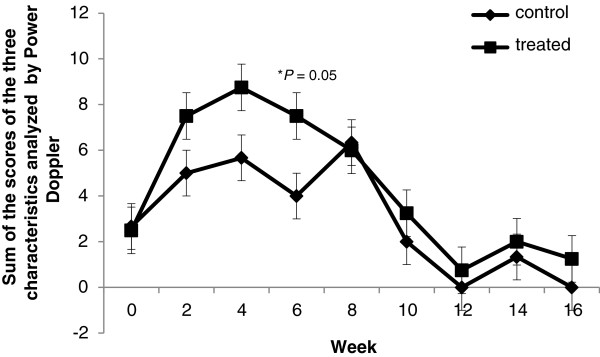
**Power Doppler evaluations.** Relationship between the Power Doppler scores in the treated group and control group at different times point after therapy.

**Figure 6:**
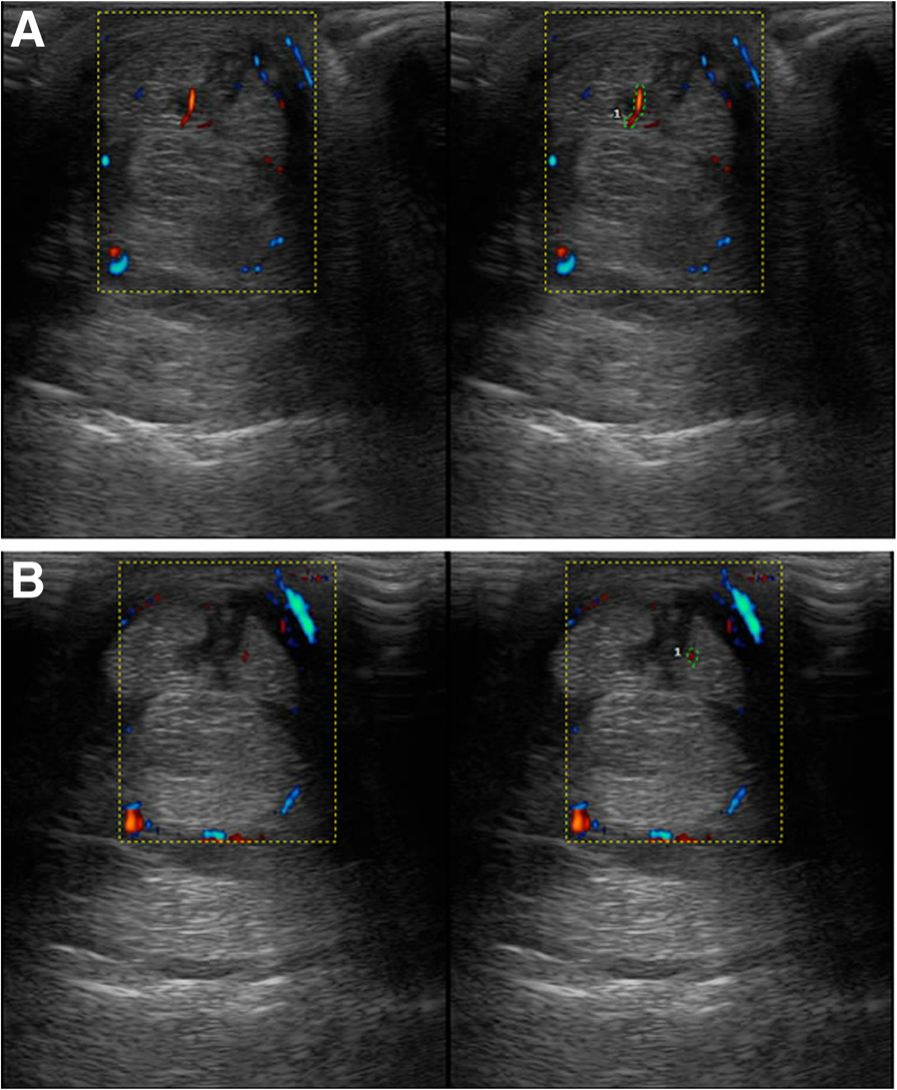
**Cross-sectional image of the Power Doppler ultrasonographic cross-sectional evaluation.** The images were obtained 12 cm distal to the accessory carpal bone six weeks after therapy. **A)** Treated group: vessel area = 3 mm^2^, signal strength = 2, number of vessels = 1. **B)** Control group: vessel area = 1 mm^2^, signal strength = 1, number of vessels = 1.

The analysis of the sum of the scores used for the evaluation of Power Doppler revealed no significant difference between the treatments (*P* = 0.097). However, when the different assessed time points were individually compared, an increased blood flow was observed in the TG compared with the CG at Week 6 (*P* = 0.05; Figures 5 and 6).

### Histopathological evaluation

**Figure 7:**
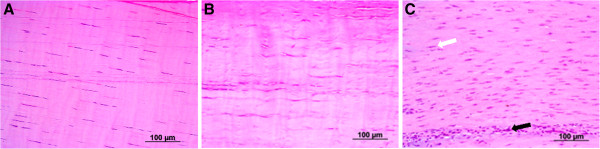
**Histopathological image of the superficial digital flexor tendon.** The figures show histopathological specimens stained with hematoxylin & eosin (H&E) using a 20× objective. **A)** Healthy group: healthy tendon. **B)** Treated group: tendon treated with the combination of MSCs derived from adipose tissue and platelet concentrate. **C)** Control group: tendon treated with PBS (the white arrow indicates the presence of a tenocyte and the black arrow indicates the presence of inflammatory cells).

The longitudinal histopathological specimens stained with H&E and Masson trichrome were analyzed and classified according to pre-established scores. Based on the analysis method used, a normal tendon would obtain a score of 8, whereas a tendon with maximum-severity histopathological damage would obtain a score of 32. The sum of the scores in the TG (14.75 ± 2.98) was significantly lower (*P* = 0.049) than the sum of the scores in the CG (22.66 ± 2.51). Overall, the TG exhibited better score results of the linearity of the collagen fibers (*P* = 0.04), compared with the CG. Among the individual variables assessed in the histopathological evaluation, the presence of inflammatory cell infiltrates showed the largest difference between the groups (*P* = 0.03): the treated group (mean = 2 ± 2.0) exhibited better results compared with the control group (mean = 3.66 ± 0.57; Figure 7).

### Immunohistochemical evaluation

The distribution and intensity of the labeling of collagen type III and factor VIII were scored in the cross-sections. No significant differences were observed in the scores given to the marking of collagen type III and factor VIII between the different groups (see Additional file 2: Figure S2).

### RNA purification and qRT-PCR

The RNA quality obtained from all of the tendon samples from all of the groups was assessed by a spectrophotometer at 260:280 nm (data not show). The RNA in the samples exhibited median concentrations of 161.85 ± 54.74 ng/μL in the TG, 132.4 ± 90.1 ng/μL in the CG, and 67.35 ± 31.05 ng/μL in the HG. The evaluation of the gene expression found no difference in the expression of the anabolic genes (*COL1A1*, *COL3A1* and *TNC*) between the different groups, but a trend higher (*P* = 0.08) expression of *COL3A1* was observed in the control group. There was no difference in the expression of the phenotypic genes (*SCX* and *TNMD*) between the different groups (see Additional file 3: Table S1).

## Discussion

The histopathological evaluation revealed that the administered therapy resulted in a better organization of collagen fibers and a decreased inflammatory infiltrate. The ultrasound evaluation showed a lack of lesion progression (lesion area). An increased number and intensity of the signals detected by the Power Doppler examination was observed; this result evidenced the presence of increased blood flow in the treated area of the TG. The therapy used did not result in any differences in the expression levels of any of the genes tested.

Tendon lesions can be induced by different methods. These techniques are basically divided into physical and enzymatic methods, such as the use of collagenase [4,5,8,13], collagenase gel [24,26], and surgical induction [30]. Although several studies have been conducted on the subject, there is still no consensus on which is the best induction technique [31]. The tendon lesion resulting from the use of collagenase for the induction of tendonitis is not identical to the lesion resulting from the etiopathogenesis of a naturally occurring injury. However, the collagenase method exhibits several advantages over the other methods, such as the development of hypercellularity, the loss of organization of the extracellular matrix (ECM), the increased vascularization, and the absence of inflammation mediated by inflammatory cells in the injured tendon [32]. The results obtained from the induction of the tendon lesion using collagenase gel are consistent with those reported previously [24]: it was possible to create consistent lesions, and there was minor extravasation of the enzyme to the epitendon.

The collection of adipose tissue, although considered an invasive procedure, proved to be a relatively simple technique [4,5,23], and no postoperative complications were observed in the animals. The isolation of the stromal vascular fraction and the subsequent culture of adMSCs until a sufficient amount was obtained (approximately 10 × 10^6^ MSCs) for the implementation of the stem cell therapy were successfully achieved during the three week period used in this experiment. Although the quantification of the cell duplication of adMSCs was not performed in the present study, the data obtained are consistent with the results previously described in studies that reported the rapid growth of these cells in culture, which enables the generation of a higher concentration of progenitor cells in a shorter period of time [17,33].

Some studies that use MSCs derived from bone marrow for the therapy of equine tendonitis use a marrow puncture to isolate the bone marrow supernatant to suspend the stem cells at the time of the therapy. There are reports of beneficial effects from this type of therapeutic combination due to the improvement in the therapeutic effect of the progenitor cells that is induced by the stimulant effects of the bone marrow supernatant [34]. In addition to the bone marrow supernatant [15,35], progenitor cells have been previously suspended in PBS [3,4,13], cell culture medium [26], autologous serum [5,10], plasma [6], and platelet-rich plasma [11]. In the present study, we chose to prepare the stem cell suspension in PC to combine the therapeutic effects of MSCs derived from the adipose tissue with the growth factors that are released by platelets after their activation. Our use of double centrifugation for the collection of the PC proved effective and enabled the collection of a PC with an average platelet number that was greater than that reported by previous studies [14,25].

Although the PC was not activated in this study, there is clinical evidence that demonstrates an improvement in tendon repair after therapy using a non-activated PC [12]. The positive results obtained with the use of non-activated PC can be explained by the *in situ* activation of platelets and the subsequent release of growth factors due to its stimulation by the components present in the local environment (for example, collagen) [36]. The activation of platelets can be induced by several methods. A recent study in horses showed that CaCl_2_ induced the highest concentration of growth factors compared with the other various types of platelet activators tested. However, we chose to not exogenously activate the platelet concentrate prior to the tendon therapy due to our uncertainty of the safety of its use *in vivo*[18].

The therapy adopted in this study, which was followed by rest with gradual increases in physical activity, was proven feasible. There were no episodes of lameness or sensitivity in the tendon after the administration of the therapy. Some researchers believe that physical activity, when correctly and gradually applied, can result in decreased tendon recovery time [11].

The results of the clinical assessment are consistent with the results obtained in previous studies: there was no difference in the sensitivity and the average circumference of the central portion of the metacarpal region between the groups [5]. There was also no difference in the degree of lameness between the groups [26]. The ultrasound evaluation revealed improvement of the cross-sectional lesion area and its percentage in the TG compared with the CG. This result is consistent with previous studies that used MSCs for the therapy of equine tendonitis [8,10,37]. The lack of significant differences between the cross-sectional lesion area and its percentage at different time points after the therapy can be justified by the correction of the *P*-values resulting from Tukey’s test and by the accidental reduction of the size of the CG.

The interpretation of the evolution of the cross-sectional lesion area and its percentage in the TG and the CG between the time of therapy (Week 0) and the subsequent assessment times shows significant differences between the groups at Weeks 4 and 6. This result demonstrated that the therapy used in this study exhibited a preventive action on the progression of the lesion because the TG showed no increase in the cross-sectional lesion area or its percentage (Figure 3).

This absence of lesion progression can be largely explained by the anti-inflammatory and immunomodulatory action of the therapy [38,39]. We hypothesize that the moment of therapy administration may have contributed to these data because the preventive action of the therapy on the progression of the tendon lesion in the TG can be more clearly demonstrated in the ultrasound evaluation (the tendon lesion is not yet fully developed at this point). If the therapy had been performed four weeks after the induction of the lesion, we would certainly not find this preventive action because the lesion would have already reached its maximum size. This statement is based on a recent study that used a technique similar to the induction of tendon injury with the collagenase gel that was used in the present study. The study demonstrated that the maximal cross-sectional area of the lesion and the maximal relative cross-sectional area of the lesion of the SDTF are found four weeks after lesion induction [24].

The use of Color Doppler ultrasound for the assessment of the vascularization in equine tendonitis [40] and after therapy for equine tendonitis with PC [19] has been described. Although Power Doppler does not provide information on the blood flow speed or direction, as can be obtained with the Color Doppler ultrasound, Power Doppler has a greater sensitivity to the presence of blood and to the blood volume, which enables the visualization of smaller vessels [27]. The results of the assessment of the vascularization obtained in this study indicate that there was increased blood flow in the treated group six weeks after the start of the therapy compared with the control group (*P* = 0.05). This result suggests that a biopsy at Week 6 would more likely find a larger number of vessels in the treated group than in the control group; this would be true in both the histopathological analysis and the immunohistochemical evaluation using factor VIII. The vessels are poorly visualized with the Color Doppler evaluation of a healthy digital flexor tendon. Hypervascularity is expected during the tissue repair process, but there is decreased vascularity with the progress of tendon repair [40].

The semi-quantitative morphological evaluation demonstrated the histopathological improvement of the treated group compared to the control group; this improvement is based on the cumulative sum of histological scores (*P* = 0.049). This evaluation also demonstrated a reduction in the inflammatory infiltrate and an improved organization of the ECM. These data are in agreement with previously reported results [4,5,13]. An improved linearity and uniformity of the collagen fibers was also observed in the treated group compared with the control group.

The action of the anti-inflammatory therapy is evident after combining the analysis of the histopathological data with the ultrasound evaluation (Weeks 2, 4 and 6). The cross-sectional area and its percentage did not worsen with therapy in the TG (Weeks 2, 4 and 6), whereas there was an increase in the lesion in the same period in the CG. This result suggests a possible anti-inflammatory effect on the progression of the lesion that prevents it from increasing in size by either minimizing or stabilizing the degeneration of the tendon fibers. The decreased inflammation after the implantation of adMSCs is likely due to the immunosuppressive effect of these cells [39,41,42]. There are several mechanisms that compose the anti-inflammatory effects of MSCs that benefit tendon repair; these include an increase in chemokines, the suppression of cytokine secretion from dendritic cells, and a reduction in the effects of T lymphocytes and natural killer cells. These data highlight the potent anti-inflammatory and immunosuppressive effects of these cells. Given these potent immunomodulatory effects of MSCs, it is not surprising that these cells are being used in clinical studies of graft-versus-host disease, which is usually a fatal condition after organ transplantation [43].

The immunohistochemical evaluation found no difference in the expression of collagen type III and factor VIII between the different groups. Previous studies have described the use of mononuclear cells derived from adipose tissue and adMSCs for the therapy of equine tendonitis. These studies have demonstrated a decreased expression of type III collagen in the treated group [4,5]. In addition, another study described the quantification of the blood vessels present in the SDFT treated with PC using factor VIII [19]: a greater amount of vessels was found in the group that received therapy with a platelet concentrate. Factor VIII is a clotting pro-cofactor that is found only on intact and functional endothelial cells of blood vessels; thus, this factor is an important tool for the evaluation of tissue vascularization [44].

The increased expression of the *COL1A1, COL3A1, TNC, TNMD* and *SCX* genes in the tendons of the TG and the CG compared with the HG are consistent with the results obtained in a previous study [24]. Soon after tendon injury occurs, the expression levels of *COL1A1* and *COL3A1* increased [45]. The expression level of *TNC*, which is an ECM protein that is synthesized during inflammation and tissue remodeling [46] and modulates the binding of cells to ECM components, also increased [47]. *TNMD* regulates the proliferation of tenocytes and the maturation of collagen fibers [48]. The increased expression of *SCX* in the injured tendons was consistent with the attempt to repair the tissue because this gene regulates the tendon progenitor cells [49] and is a marker of tendon and ligament development [49].

Although an increases expression of the tested genes was observed in the injured tendons (TG and CG) compared with the healthy tendons (HG), no difference was observed between the expression in the different injured groups (TG and CG). This result is consistent with the results obtained in previous studies [13,26]. However, a trend for increases in the expression of *COL3A1* was observed in the control group (P = 0.08). Soon after tendon injury, the gene expression levels of collagen type I and III increase. The decrease in the concentration of collagen type I compared with that of collagen type III is more closely related to the formation of scar tissue than to tendon regeneration [26]. Thus, the lower expression of the *COL3A1* gene in the treated group suggests that the tendons in the TG are better repaired than those in the CG. However, this result should be examined with caution because the difference in the expression levels of *COL3A1* between the groups was not significant and because no increase in the collagen type III protein expression was observed in the immunohistochemical evaluation.

Only one study has reported differences in gene expression after the use of cell therapy for equine tendonitis. This study reported an increase in the expression of the gene cartilage oligomeric matrix protein (*COMP*) after tendonitis therapy with mononuclear cells derived from adipose tissue [4]. A likely explanation for the lack of gene expression differences between the tendons of the TG, which exhibited improved histological and ultrasound characteristics, and the tendons of the CG is that the tested genes, although associated with tendon repair, are not specific. Thus, further research is necessary to select genes that are better markers of tendon repair [50,51].

A limitation of the present study was the fact that the investigation did not determine the exact mechanism of action of the therapy that was used. This lack could have been due to trophic action (local modulation of cytokines), the replacement of cells from the injured tissue, local immunomodulation, or other mechanisms that favor the improvement of tissue repair evidenced by histopathological and ultrasound analyses.

There is great difficulty in the development of a well-designed large-scale study involving equines. The lack of basic knowledge of the behavior of adult stem cells and PC after their administration is also undeniable. Progenitor cells are believed to differentiate in the specific tissue site into which they were deployed (for example, tenocyte differentiation) and thus promote the production of the appropriate ECM. These cells can also synthesize bioactive proteins (for example, growth factors and cytokines) that promote the recruitment of endogenous stem cells and the anabolic stimulation of newly recruited cells and the mature cells of the tissue itself [16].

Similar to other experiments that used cell therapy with or without PC for the treatment of equine tendonitis [4-8,11,13,26], the present study demonstrated a satisfactory result. However, it has limitations regarding the effects of the therapy used. The results demonstrate that there is no definition for the effectiveness of a therapy for the treatment of equine tendonitis. Based on the preventive result obtained with the adMSCs suspended in PC on the progression of the lesion in the ultrasound evaluation (cross-sectional lesion area and its percentage), further studies should be conducted to confirm this preventive action and the mechanism of action of the therapeutic association, as well as the action of progenitor cells and platelets alone as the lesion progresses. This conclusion is consistent with that described in a recent study on tendonitis, which states that prevention can have a significantly greater clinical impact than therapy due to the impossibility of tendon regeneration [52].

Future studies should be conducted to further elucidate the ideal time of administration of the equine tendonitis therapy, which is currently standardized between 7 and 45 days after the onset of the lesion. Additionally, further studies would contribute to the understanding of when the best therapeutic results are obtained and whether it really is possible to prevent further injury when the therapy is administered to a still-developing injury.

Basic studies that elucidate the real mechanism of action of the therapy used in tissue repair should also be conducted to confirm the differentiation of the stem cells used, analyze the release of trophic factors (for example, cytokines and growth factors), and verify the anti-inflammatory and immunomodulatory action of the therapy. Better knowledge of the etiopathogenesis of tendonitis associated with the knowledge of the mechanism of action of adult stem cells and activated platelets will enable improved clinical outcomes and result in the verification of the therapeutic efficacy of this new biotechnology.

## Conclusions

The present study demonstrated that the use of adMSCs combined with PC had a favorable action in the therapy of tendonitis. This result was verified mainly by the prevention of the progression of the lesion, which was observed in the ultrasound evaluation, and by the significant improvement observed in the histopathological analysis scores. Future studies should be conducted to elucidate the immunomodulatory action of adMSCs combined with PC and the best time for its administration.

## Abbreviations

ACB: Accessory carpal bone; adMSCs: Mesenchymal stem cells derived from adipose tissue; CG: Control group; COL1A1: Type I collagen; COL3A1: Type III collagen; COMP: Gene cartilage oligomeric matrix protein; CT: Threshold cycle; DMEM: Dulbecco’s modified Eagle’s medium; ECM: Extracellular matrix; FBS: Fetal bovine serum; H&E: Hematoxylin and eosin; HG: Healthy group; MSCs: Mesenchymal stem cells; P1: First pass; PBS: Phosphate buffered saline; PC: Platelet concentrate; PRF: Pulse repetition frequency; RT-PCR: Real-time polymerase chain reaction reverse transcription; SCX: Scleraxis; SDFT: Superficial digital flexor tendon; TG: Treated group; TNC: Tenascin-C; TNMD: Tenomodulin.

## Competing interests

The authors declare that they have no competing interests.

## Authors’ contributions

AMC, ALGA, ASB, CAH and ED contributed to the conception and design of the study. AMC, PRB, LECA and ALMY collected and assembled the data. AMC, PRB, LECA, ALMY, ASB, CAH, ED and ALGA took part in data analysis and interpretation. AMC, ALMY, PRB, LECA and ALGA wrote and revised the paper, while AMC, ALGA, ASB and ED edited the manuscript. ALGA and CAH were in charge of financial and administrative support. All authors have read and approved the manuscript for publication.

## Supplementary Material

Additional file 1: Figure S1 Biopsy of the superficial digital flexor tendon (SDFT). **A)** Isolation of SDFT in the central metacarpal region of forelimb, 10 to 11 cm distal to the accessory carpal bone. **B)** Biopsy of the lesion area in the center of the SDFT. Click here for file

Additional file 2: Figure S2 Immunohistochemical image of the superficial digital flexor tendon. **A)** Immunohistochemical image for collagen III, treated group, 40× objective. **B)** Immunohistochemical image for collagen type III, control group, 40× objective. **C)** Immunohistochemical image for factor VIII, treated group, 20× objective. **D)** Immunohistochemical image for factor VII, control group, 20× objective. Click here for file

Additional file 3: Table S1 Gene expression. Median of the gene expression levels in the different groups relative to the gene expression levels obtained in healthy tendons. Click here for file
