# Supplementary figures and images for: Equine tendonitis therapy using mesenchymal stem cells and platelet concentrates: a randomized controlled trial
Source: Stem Cell Res Ther. 2013 Jul 22;4(4):85. doi: 10.1186/scrt236 (PMC3854756; doi:10.1186/scrt236)

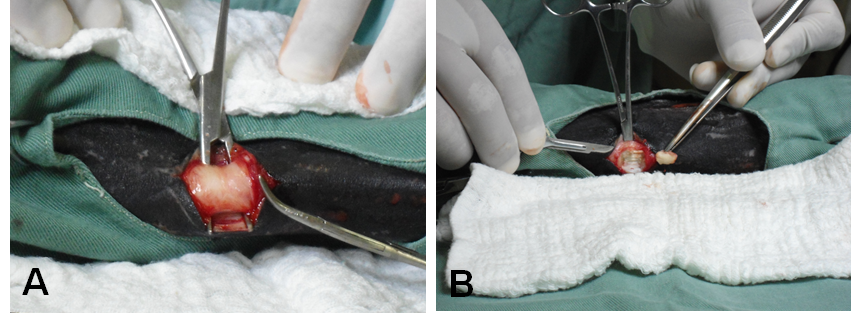

Supplement: Additional file 1: Figure S1 — Biopsy of the superficial digital flexor tendon (SDFT). A) Isolation of SDFT in the central metacarpal region of forelimb, 10 to 11 cm distal to the accessory carpal bone. B) Biopsy of the lesion area in the center of the SDFT. [file scrt236-S1.tiff]

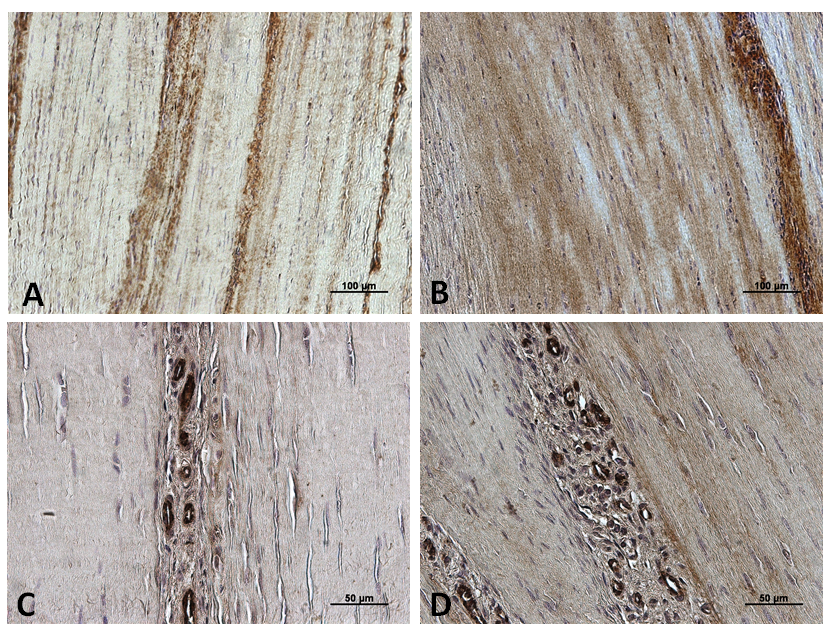

Supplement: Additional file 2: Figure S2 — Immunohistochemical image of the superficial digital flexor tendon. A) Immunohistochemical image for collagen III, treated group, 40× objective. B) Immunohistochemical image for collagen type III, control group, 40× objective. C) Immunohistochemical image for factor VIII, treated group, 20× objective. D) Immunohistochemical image for factor VII, control group, 20× objective. [file scrt236-S2.tiff]
