# Supplementary material for: Equine tendonitis therapy using mesenchymal stem cells and platelet concentrates: a randomized controlled trial
Source: Stem Cell Res Ther. 2013 Jul 22;4(4):85. doi: 10.1186/scrt236 (PMC3854756; doi:10.1186/scrt236)
Supplement: Additional file 3: Table S1 — Gene expression. Median of the gene expression levels in the different groups relative to the gene expression levels obtained in healthy tendons. [file scrt236-S3.doc]

| Gene | Treated Group | Control Group |
| --- | --- | --- |
| *COL1A1* | 38.90 | 69.95 |
| *COL3A1* | 92.59* | 390.60^*P=0.08^ |
| *SCX* | 10.40 | 14.37 |
| *TNMD* | 1034.15 | 1318.31 |
| *TNC* | 6.99 | 8.62 |
